# Prevalence of Polypharmacy and Inappropriate Medication in Adults With Intellectual Disabilities in a Hospital Setting in Switzerland

**DOI:** 10.3389/fpsyt.2021.614825

**Published:** 2021-06-25

**Authors:** Sophie Lonchampt, Fabienne Gerber, Jean-Michel Aubry, Jules Desmeules, Markus Kosel, Marie Besson

**Affiliations:** ^1^Psychopharmacology Unit, Division of Clinical Pharmacology and Toxicology, Department of Anesthesiology, Intensive Care, Pharmacology and Emergency, Geneva University Hospitals, Geneva, Switzerland; ^2^Unit for Treatment and Assessment of In and Out Patients With Learning Disabilities and Autism Spectrum Disorders, Division of Psychiatric Specialties, Department of Psychiatry, Geneva University Hospitals, Geneva, Switzerland; ^3^Faculty of Science, School of Pharmaceutical Sciences, University of Geneva, Geneva, Switzerland; ^4^Division of Psychiatric Specialties, Department of Psychiatry, Geneva University Hospitals, Geneva, Switzerland; ^5^Division of Clinical Pharmacology and Toxicology, Department of Anesthesiology, Intensive Care and Pharmacology, Geneva University Hospitals, Geneva, Switzerland

**Keywords:** intellectual disability, polypharmacy, inappropriate prescription, psychotropic drugs, deprescription

## Abstract

**Background:** Polypharmacy and inappropriate prescription are frequent in vulnerable and multi-morbid populations. Adults with intellectual disability (ID) are at risk of being polymedicated because they often present with multiple comorbidities and challenging behaviors.

**Aim:** The objective of this study was thus to evaluate the prevalence of potentially inappropriate medications (PIM) and polypharmacy in a hospital unit dedicated to adults with ID.

**Methods:** A 10-month prospective observational study took place at a hospital unit specializing in the care of adults with ID in Geneva, Switzerland. Once a week, health and prescription data were collected and screened for PIM according to preset definitions.

**Results:** Fourteen patients consented to participate, leading to 20 hospitalization events assessed during the study. Hospitalizations lasted 12.8 weeks on average. ID severities ranged from mild to profound, all degrees of severity being equally represented. One hundred percent of the patients were polymedicated (defined as five drugs or more prescribed simultaneously). A mean number of 9.4 drugs were prescribed per week, including 5.3 psychotropic drugs. The number of prescribed drugs remained stable throughout the hospitalizations. Antipsychotics were the most prescribed drug class (19% of all prescribed drugs), followed by benzodiazepines (13%) and laxatives (12%). A total of 114 PIM were recorded with an average of 5.7 PIM per hospitalization.

**Conclusions:** This study showed that polypharmacy and inappropriate prescription are very common in adults with ID, even though the literature and expert positions advocate for deprescription in these patients. Specific prescribing and deprescribing guidelines are needed for that specific population.

## Introduction

Among the numerous definitions of polypharmacy, the most used is the concurrent use of five or more medications (1). Polypharmacy can be appropriate when drugs are prescribed for patients with multiple comorbidities in a way to improve quality of life or prognosis or even minimize drug side effects (2). However, polypharmacy often is the consequence of inappropriate prescribing and prescription errors and leads to an increased risk of adverse events (2–4). In a population-based study, the risk of an adverse drug event increased by 88% for patients taking five drugs or more compared to those who were taking one or two drugs (5).

Studies in vulnerable and polymedicated populations, such as the elderly, showed that inappropriate prescriptions are frequent. The prevalence of inappropriate prescriptions varied between 24 and 73% in a study conducted on geriatric outpatients under polypharmacy (6). Such prevalent numbers vary greatly because medication appropriateness is determined by different criteria. Appropriateness criteria are either explicit [e.g., Beers criteria (7)] or implicit [e.g., Medication Appropriateness Index (8)]. Examples of explicit criteria are the occurrence of expected side effects of prescribed drugs such as falls or confusion in the elderly. Implicit criteria however may consider drug indication, dosage, duration, interactions, or duplications.

The population of adults with ID is at risk of polypharmacy and inappropriate prescribing due to multiple comorbidities, a high prevalence of challenging behaviors, and the lack of specific prescribing guidelines. Psychotropic drugs in particular are highly prescribed in that population, sometimes without efficacy and with drug–drug interactions leading to numerous adverse drug effects (9–11). A study by Haider et al. (12) in Australia showed that the prevalence of polypharmacy in that population was about 21%. Prescription in adults with ID is further complicated by the fact that they are systematically excluded from clinical trials assessing medication, and as a result indication and benefit–risk balance are rarely clearly determined.

There have been very few studies of drug appropriateness in people with ID. However, a Dutch study found a total of 127 drug-related problems in 27 adults with ID, mainly the prescription of potentially unnecessary or inappropriate drugs (13).

The objective of this study was thus to evaluate the prevalence of polypharmacy and inappropriate medication (PIM) in a hospital unit dedicated to adults with ID in Geneva, Switzerland.

## Methods

### Setting, Design, and Study Population

This was a prospective observational study, which took place between February 2019 and December 2019 at the unit for adults with ID in the Geneva University Hospital, Switzerland. About 150 hospitalization events (corresponding to about 90 different patients) take place each year at the unit which offers care for 18 in-patients.

Any patient who was admitted at the unit during the duration of the study, or his legal representative, was asked to participate. Simplified oral and written information was given to the patient when possible and oral and written information was offered to their legal representative and to the patient when possible. Written informed consent was signed by the patient or the legal representative.

For each patient, the follow-up period lasted for the whole duration of hospitalization or until the end of the study period. If a patient was hospitalized more than once during the study period, each hospitalization was recorded as a separate event.

The authors assert that all procedures contributing to this work comply with the ethical standards of the relevant national and institutional committees on human experimentation and with the Helsinki Declaration of 1975, as revised in 2008. All procedures involving patients were approved by the Ethics Committee of Geneva (approval number: 2018-01790).

### Measures

After inclusion, demographic data, and comorbidities were recorded, as well as prescription data, potential adverse events, and scores of three psychometric scales (the Aberrant Behavior Checklist (ABC) (30), the Health of the Nation Outcome Scales for Learning Disabilities [HoNOS-LD) (31), and the Clinical Global Impression scale (CGI) (32)].

The ABC scale is widely used to assess the presence and severity of challenging behaviors in patients with ID, as well as treatment efficacy. It consists of 58 items graded on a four-point scale, from 0 (the behavior is not a problem at all) to 3 (it is a significant problem), spread over five factors: irritability, agitation (F1–15 items), social withdrawal (F2–16 items), stereotyped behaviors (F3–7 items), hyperactivity/noncompliance (F4–16 items), and inappropriate language (F5-4 items) (30). The higher the score, the greater the behavioral problem. The ABC was completed for each participant by a clinician.

The CGI is a short scale used to objectify the severity and the improvement of the patients' clinical status. It is a brief assessment of the clinician's view of the patient's global functioning prior to and after initiating a pharmacological treatment. The CGI scale takes the therapeutic effect and the presence of side effects into account. The scale comprises four items: the illness severity score (1–7), the global improvement score (1–7), the side effects score (1–4), and the therapeutic effect score (1–4). The CGI index (1–8) is the sum of the side effects score and the therapeutic effect score. The lower the index score, the greater the therapeutic effect and the lower the side effects (32).

The HoNOS-LD is adapted from the Health of the Nation Outcome Scale (HoNOS), a tool to evaluate symptom severity and social functioning among adults with mental health problems (33). The HoNOS-LD is a specific version of the HoNOS developed for individuals with ID. HoNOS-LD has 18 items rated on a scale from 0 to 4. It measures disability, behavior, impairment, symptoms, and social functioning (31). The total score is the sum of all the 18 items (0–72). The higher the score, the greater the mental health problem.

Once a week, health status, potential adverse events, and prescription data were collected from the hospital electronic database by the study investigator. The data extracted concerned drug prescriptions (posology, duration, and indication), potential adverse events based on the patient symptoms, and the patient clinical evolution as reported by the caregivers. The prescription data were then screened for appropriateness; the number and type of potentially inappropriate medications (PIM) as defined in Table 1 were recorded.

**Table 1:** Potentially inappropriate medications (PIM) were defined as a prescription that meets one of the listed criteria [adapted from (14, 15)].

**PIM criteria**	**Description and examples**
Lack of indication	A treatment which has no indication according to the clinical context of the patient^*^
Unwanted pharmacokinetic interaction	e.g., a CYP2D6 inhibitor prescribed with a CYP2D6 metabolized drug
Unwanted pharmacodynamic interaction	e.g., prescription of two anticholinergic drugs
Unwanted drug–disease interaction	Drugs to avoid in patients with specific conditions, e.g., ibuprofen prescribed to a patient with renal failure
Duplicate (or more) prescribing	e.g., prescription of two antipsychotics
Incorrect prescription duration	e.g., a benzodiazepine prescribed for more than 4 weeks
Incorrect use	e.g., abrupt withdrawal of an antipsychotic
Lack of documentation	Lack of indication in the patient's file on why the drug has been prescribed

* *Concerning psychotropic drugs to treat challenging behaviors, there are no evidence-based guidelines. However, based on some data and expert recommendations in the literature, we decided to consider the following treatments as appropriate for the treatment of challenging behaviors: risperidone (16–18), aripiprazole (19–21), olanzapine (17, 22), quetiapine (22), clozapine (23), zuclopenthixol (16), haloperidol (24–26), and lithium (27) for the treatment of aggression and irritability, and naltrexone (24, 28, 29) for the treatment of self-injurious behaviors*.

Adverse effects were not considered in the criteria of PIM and were recorded separately. Drug imputability was assessed according to the WHO-UMC classification.

**Figure 1:**
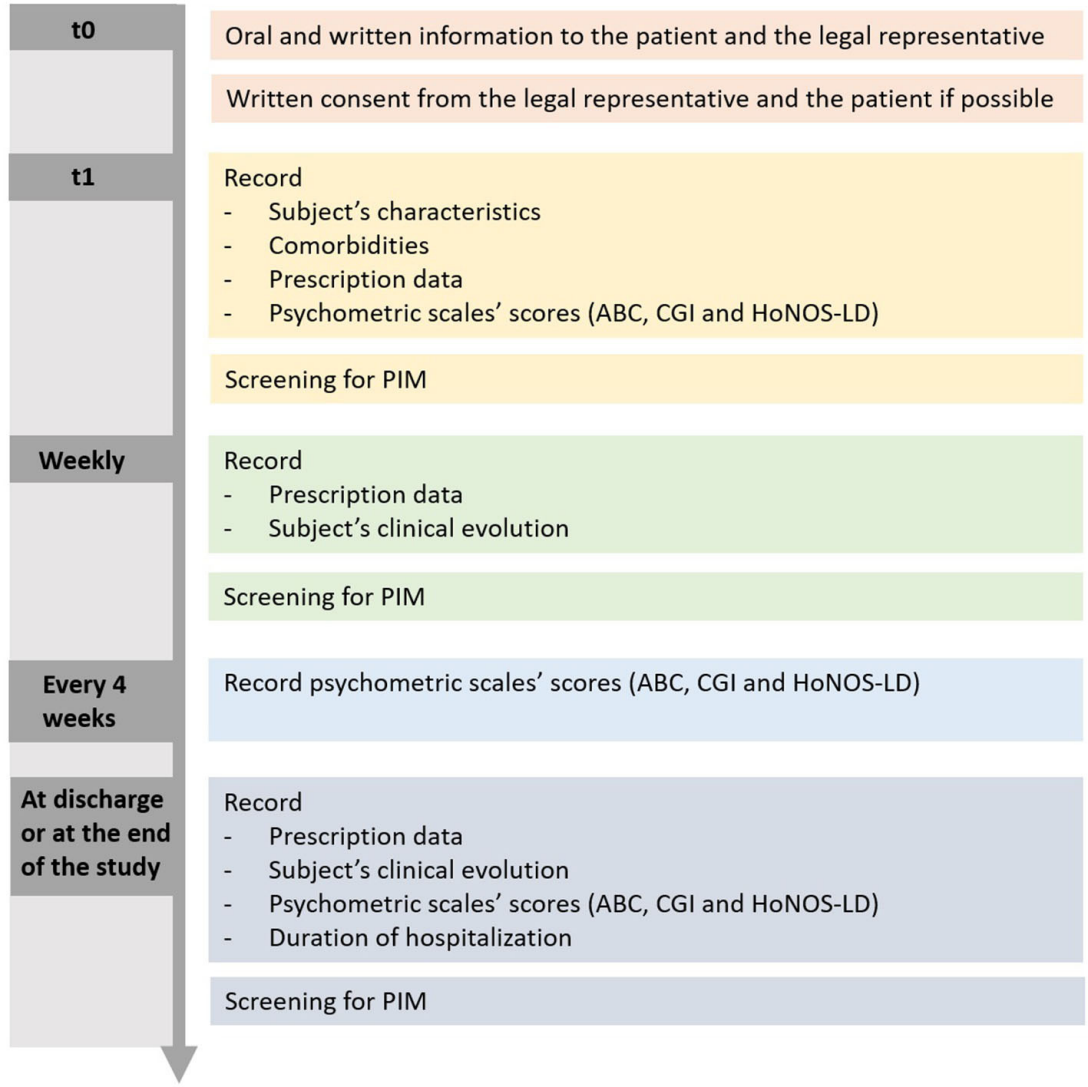
Typical schedule of assessment for a patient.

The ABC, CGI, and HoNOS-LD scores were recorded on a monthly basis and at the end of the stay. Figure 1 summarizes the typical schedule of assessments for a patient included in the study.

All drugs were recorded except vitamins, food supplements (e.g., magnesium, folate, calcium), lotions, creams, or eye drops. Rescue drugs were included in the prescription data as they were frequently given to the patients.

### Data Analysis

Data were analyzed using the IBM SPSS Statistics 25 software. Descriptive statistics were used to summarize comorbidities, prescription data, and PIM data. Two-tailed paired samples *t*-tests were used to analyze the evolution of psychometric scores and treatment numbers over time. Pearson correlation and *t*-test for equality of means were used to analyze associations between treatment numbers or PIM numbers and health or demographic data such as psychiatric diagnoses, ID severity, age, and sex.

## Results

### Demographics

Of the 65 hospitalization events, corresponding to 50 screened patients, 14 consents were obtained (13 by the legal representative and 1 by the patient himself). This corresponds to 20 hospitalization events, as 6 patients were hospitalized twice during the study period.

The majority of patients were male (75%), and the median age was 34 years (range: 17–55 years). The median duration of hospitalization was 6.5 weeks (range: 2–45 weeks).

ID severities ranged from mild to profound, all degrees of severity being equally represented: mild *n* = 5 (25%), moderate *n* = 6 (30%), severe *n* = 5 (25%), and profound *n* = 4 (20%).

### Comorbidities

Challenging behaviors were the reason for hospitalization in 100% of the cases. Table 2 describes the types of challenging behaviors and their prevalence in the study sample.

**Table 2:** Types and prevalence of challenging behaviors in the study sample.

**Type of challenging behavior**	**Number of hospitalization events for which the behavior was present**
Agitation	8
Impulsivity	2
Verbal aggression	6
Physical aggression	15
Object destruction	5
Self-injury	8
Screams	3
Running away from the institution	1

The median number of comorbidities per hospitalization event was 4 (range 1–9), and the median number of psychiatric comorbidities was 1 (range 0–4). Table 3 describes the types and prevalence of comorbidities in the study sample. The most frequent types of comorbidities were psychiatric comorbidities (32), with childhood autism in particular with 10 occurences, followed by gastrointestinal (13) and metabolic disorders (10).

**Table 3:** Types and number of comorbidities in the study sample (on 20 hospitalization events).

**Type of comorbidity**	**Number of comorbidities present (on 20 hospitalization events)**
**Mental health**	**Total: 32**
Childhood autism	10
Depressive episode	3
Borderline disorder	4
Psychotic disorder	5
Anxiety disorder	2
Bipolar affective disorder	2
Gender identity disorder	1
Suicide attempt	1
Smoking dependence	2
Sleep disorder	2
**Nervous system**	**Total: 7**
Epilepsy	3
Motor disability	1
Parkinsonism	1
Encephalopathy	1
Developmental venous anomaly	1
**Gastrointestinal**	**13**
**Metabolic**	**10**
**Heart**	**9**
**Eye**	**5**
**Genitourinary system**	**3**
**Musculoskeletal**	**2**
**Skin**	**2**

### Psychometric Scales

Except for the ABC F2 and F3 scores and the CGI side effects score, a significant difference was found between the scores at entry in the study and at the end of the stay. Table 4 describes the median differences of the statistically significant scores. Mental health and behavioral status improved during the hospitalization.

**Table 4:** Median scores (range) of the ABC scores, HONOS-LD, and CGI at the entry and at the end of the study.

	**Entry**		**End**		**Related samples Wilcoxon rank test**
	***N***	**Median (range)**	***N***	**Median (range)**	***p***
ABC F1 irritability	20	9 (2–21)	20	2.0 (0–15)	0.014^**^
ABC F2 social withdrawal	20	7.0 (0–20)	20	2.0 (0–23)	0.064
ABC F3 stereotyped behaviors	20	1.50 (0–12)	20	1.0 (0–10)	0.107
ABC F4 hyperactivity/non-compliance	20	11.0 (3–36)	20	5.0 (0–21)	0.000^**^
ABC F5 inappropriate language	20	1.0 (0–9)	20	0.5 (0–5)	0.025^*^
HoNOS-LD total	20	20.5 (5–42)	20	15.5 (4–31)	0.003^**^
CGI illness severity	20	4.5 (2–6)	20	4.0 (1–5)	0.017^*^
CGI global improvement	20	3.0 (0–6)	20	2.0 (1–4)	0.027^*^
CGI side effects	20	0.0 (0–2)	20	0.0 (0–1)	0.218
CGI therapeutic effects	20	9.0 (1–13)	20	5.0 (1–13)	0.026^*^
CGI index	20	9.0 (1–15)	20	5.0 (1–13)	0.026^*^

* *Significance p < 0.05*;

** *significance p < 0.01*.

### Medication Analysis

#### Number of Drug Treatments

Patients were prescribed a median number of 8.5 drugs per week (range 5–14) and 5 psychotropic drugs (range 2–10). There was no statistical difference between the mean number of drugs at admission and at the end of the stay. In 45% of the hospitalization events, the number of treatments did not change between entry and end of stay. In 20% of the events, the number of treatments decreased, and in 35% of the events, it increased.

#### Polypharmacy

During all hospitalizations (100%), 5 or more drugs were prescribed and during 40% 10 or more. During 70% of hospitalizations, 5 or more psychotropic drugs were prescribed.

#### Drug Classes

**Figure 2:**
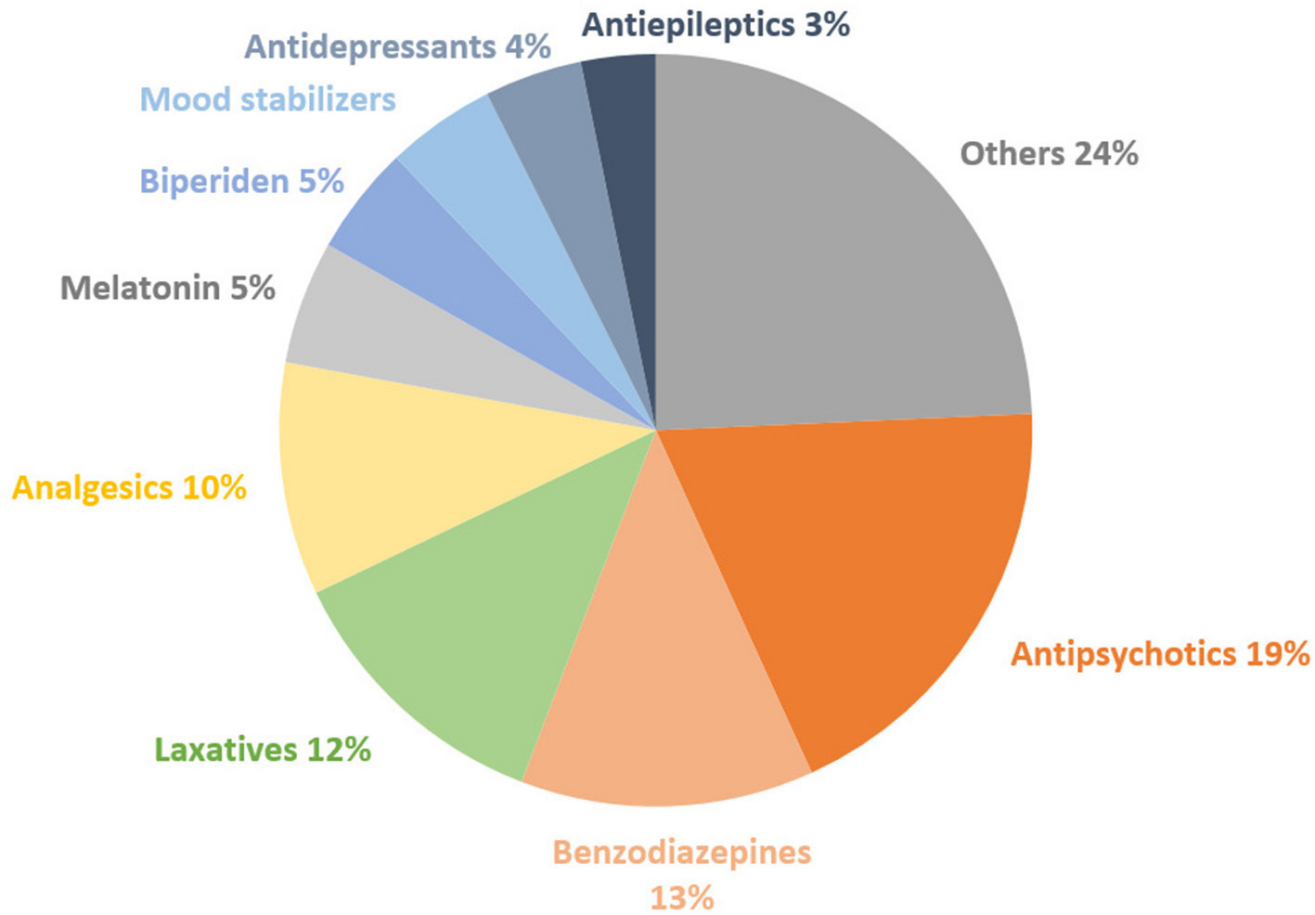
Distribution of the types of drugs on the total number of drugs prescribed to all subjects.

Figure 2 illustrates the distribution of the most prescribed drugs. Antipsychotics came first (19%), followed by benzodiazepines (13%) and laxatives (12%). Table 5 shows that during 100% of hospitalizations, at least one antipsychotic drug was prescribed. At least one analgesic drug (mainly paracetamol or ibuprofen) was prescribed during 80% of hospitalizations, at least one benzodiazepine in 75% and at least one laxative in 70%.

**Table 5:** Percentage of patients taking at least one of the drugs of the listed drug classes during their stay (on 20 hospitalization events).

**Class of drug**	**Percentage of patients taking at least 1 drug of the concerned class during their stay (on 20 hospitalization events)**
Antipsychotic	100
Analgesic	80
Benzodiazepine	75
Laxative	70
Melatonin	50
Biperiden	45
PPI (esomeprazole)	35
Antidepressant	35
Metabolic	30

No correlation was found between different psychiatric disorders (ASD, bipolar, psychosis) and the number of treatments, nor between ID severity, age or sex, and the number of treatments.

The most prescribed antipsychotics were levomepromazine (15% of the prescribed antipsychotics), risperidone (13%), aripiprazole (13%), olanzapine (13%), quetiapine (13%), and clotiapine (13%). The other antipsychotics prescribed were haloperidol (8%), zuclopenthixol (5%), clozapine (5%), and paliperidone (3%).

### PIM Analysis

**Figure 3:**
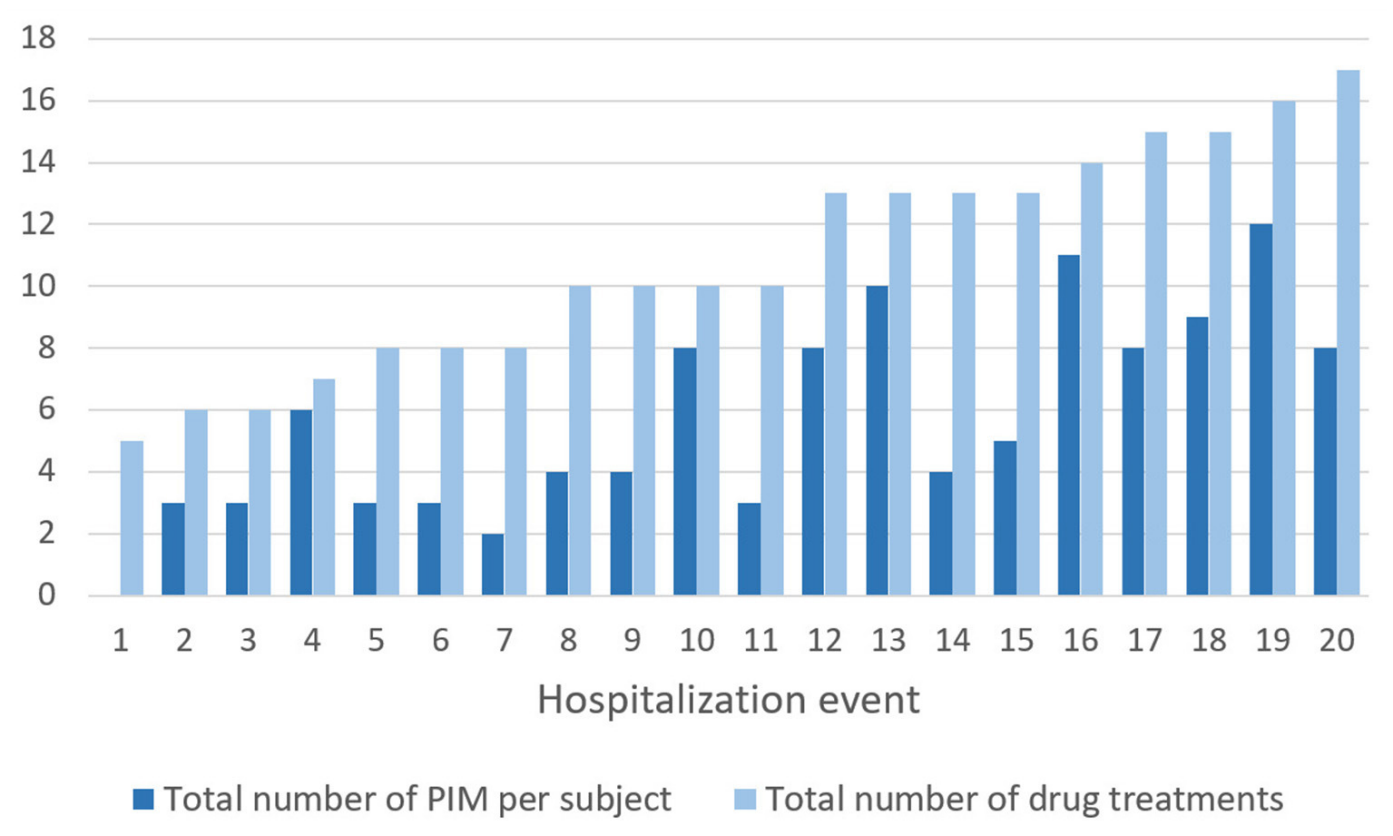
Total number of PIM and number of drugs prescribed per hospitalization event (listed in ascending order of number of treatments).

A total of 114 PIM were recorded during the study with an average of 5.7 (0–12) PIM per hospitalization. Figure 3 depicts the number of PIM and the number of drugs prescribed during each hospitalization. Fifty-two percent of the drug prescriptions were potentially inappropriate. Only one patient did not have any PIM. The difference between the mean number of PIM at admission (mean = 4.3; sd = 2.41) and at the end (mean = 4.2; sd = 2.57) was not statistically significant {*t*-test [*t*_(19)_ = 0.295; *p* = 0.772]}.

**Figure 4:**
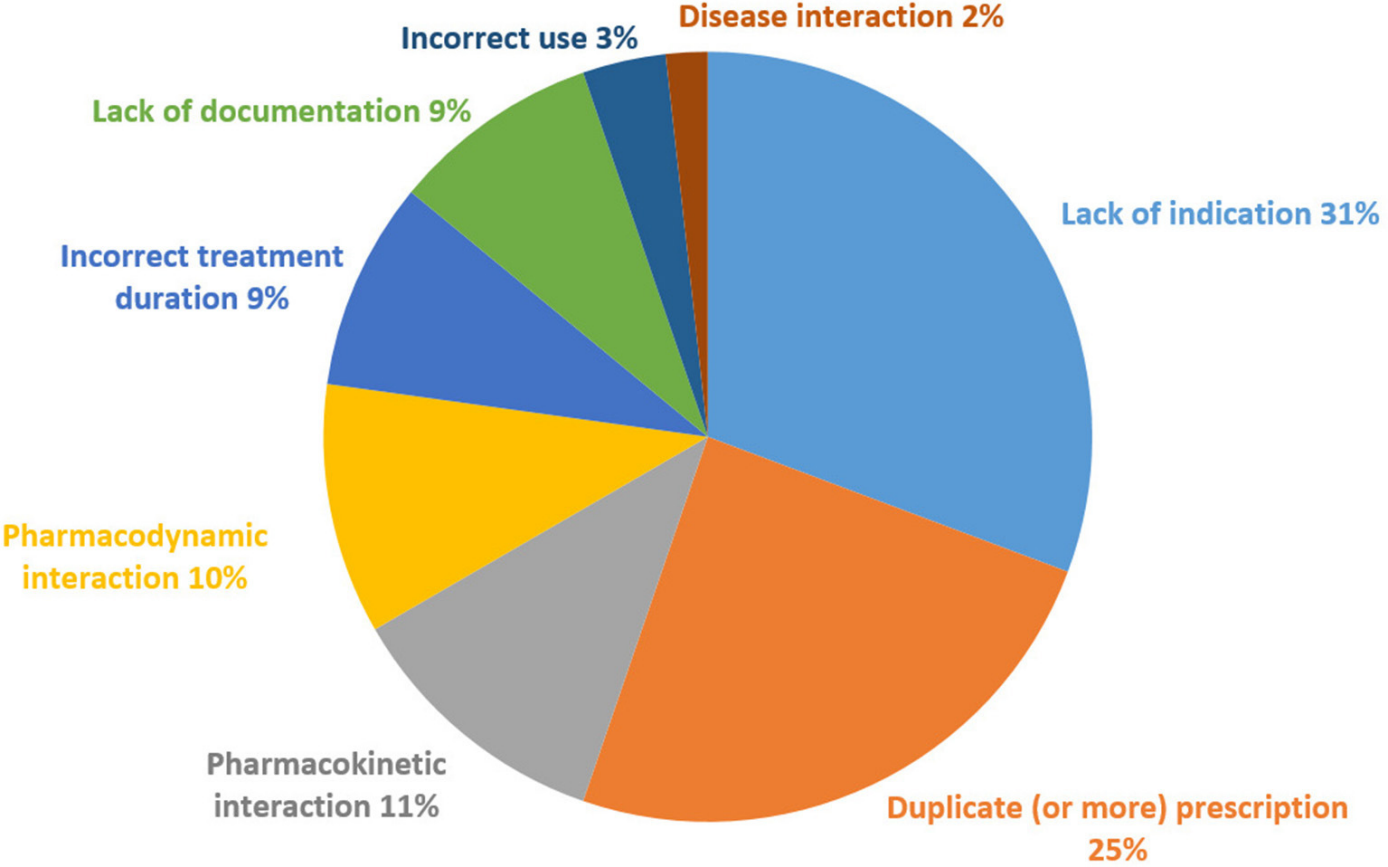
Distribution of PIM types during all hospitalizations.

Figure 4 describes the distribution of PIM types across all hospitalizations. The most common PIM type was a lack of indication (31%), followed by duplicate prescribing (25%) and pharmacokinetic interactions (11%). The correlation between the total number of PIM and the total number of drug treatments was not significant (*r* = −0.289; *p* = 0.217).

All the recorded PIM are listed in Appendix 1 along with their occurrence. Most drugs that lacked indication according to the clinical context were psychotropic drugs, namely, antidepressants. The duplicates concerned antipsychotics, benzodiazepines, and laxatives. There were two cases of triplicate prescriptions of antipsychotics, one prescription of three antiepileptic drugs, one prescription of four antiepileptic drugs, and one prescription of three benzodiazepines. The pharmacokinetic interactions mainly concerned the CYP2D6 cytochrome. The pharmacodynamic interactions mainly concerned anticholinergic drugs. The only drug class concerned by incorrect prescription duration was the class of benzodiazepines, with a prescription >4 weeks. An incorrect use mainly concerned abrupt withdrawals of psychotropic drugs.

No correlation was found between the number of PIM and the ID severity or age of the subjects. No differences were found between males and females.

### Adverse Events

**Figure 5:**
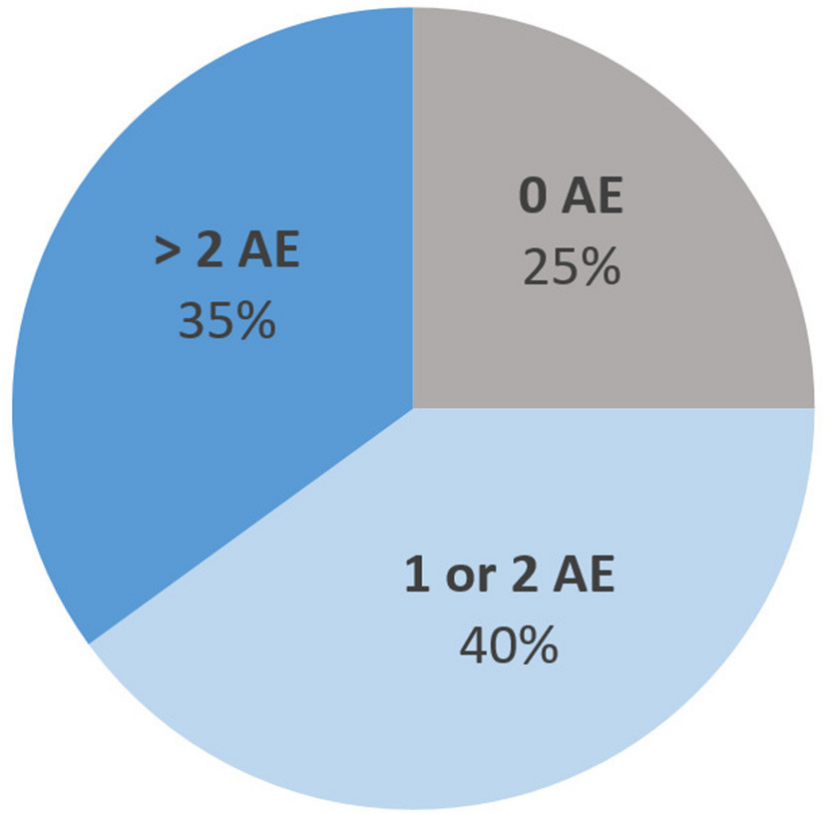
Distribution of the maximum number of concomitant adverse events (AE) per hospitalization event.

Figure 5 describes the distribution of the number of adverse events (AE). The most common types of AE were gastrointestinal AE (27% of all AE), followed by metabolic AE (20%), cardiac AE (15%), anticholinergic AE (11%), and extrapyramidal symptoms (7%). Five of the 14 subjects (36%) displayed an episode of QT prolongation during their stay (defined by a QTc > 440 ms in men and 460 ms in women). All AE were classified as “possible AE” based on the WHO-UMC classification (34), except for two AE which were classified as “probable”: hyperprolactinemia and secondary Parkinson syndrome with clotiapine and quetiapine.

## Discussion

This prospective observational study confirmed that among a population of adults with ID admitted to a tertiary dedicated hospital unit, the prevalence of polypharmacy and potential inadequate medication (PIM) was extremely high, since 100% of the included patients were polymedicated. An average of 5.7 (STD = 3.3) PIM per hospitalization event was observed. Our study population consisted mainly of young adults across all ranges of ID (mild to profound). All subjects were hospitalized because of challenging behaviors.

The mean number of drugs per hospitalization event was 9.4 (2.9) per week, which is higher than in the study by Scheifes et al. (35). In that study, the mean number of prescriptions per patient was 5.2. The study also took place in a specialized inpatient unit for adults with ID and challenging behaviors but was located in the Netherlands. This could indicate different prescribing practices for ID patients across different European countries.

The most prescribed drug classes were antipsychotics and benzodiazepines. Such drugs are often used to try to treat challenging behaviors, which is the main reason for all hospitalizations in our study. Even without an underlying psychiatric diagnosis justifying their use, they are prescribed for their unspecific sedative and anti-impulsivity effects. The most prescribed antipsychotic was levomepromazine, followed by risperidone, aripiprazole, olanzapine, quetiapine, and clotiapine. The important number of prescriptions of levomepromazine and clotiapine is a local habit (9, 36, 37). This result underlines the need for change in prescription practices as first-generation antipsychotics should not be used in the first line because of their adverse effect profile and the risk of irreversible extrapyramidal symptoms.

Among the top 10 prescribed drugs, laxatives came in third position. This is expected since constipation is frequent in people with ID and a well-known adverse effect of antipsychotics. Analgesics such as paracetamol and NSAIDS came in fourth position. Indeed, analgesics are often prescribed in the situation of challenging behaviors because they might be related to a painful condition (38). Other non-psychotropic drugs that were often prescribed in our population were proton pump inhibitors (PPIs). Again, gastroesophageal reflux is a recurrent suspected problem in adults with ID. Moreover, as in the general population, PPIs are often prescribed off-label, especially in polymedicated patients (39–42). The most frequent inappropriate uses of PPIs are the prevention of gastroduodenal ulcers in low-risk patients and the overtreatment of dyspepsia (42). Dyspepsia is in fact a common complaint in psychiatric patients, partly due to the high prevalence of polypharmacy (43).

A total of 114 PIM were identified, which corresponds to 52% of drug prescriptions. This is in line with two other studies by Zaal et al. In the first one, in older individuals with ID in an outpatient setting, the author reported that 47.5% of the subjects had one or more PIM (44). In the second one, four drug-related problems were found per subject in an outpatient setting (13). Moreover, our results are similar to those found in other vulnerable and polymedicated populations, such as the geriatric population (6).

PIM were mainly related to psychotropic drugs. The most common problem was the lack of indication, even though indications were considered in the broad sense, including usual clinical practice as well as issuing market authorizations. This was namely the case for antidepressants which were often prescribed, although no diagnosis of depressive disorder had been made. Antidepressants were prescribed in 35% of hospitalization events, whereas a diagnosis of a depressive episode or anxiety disorder was only documented in 15% of these events. This is in line with observations in the general population where antidepressants are often prescribed for an off-label use (45). Indeed, antidepressants are a class of drugs that might be considered for systematic deprescribing.

Pharmacokinetic drug–drug interaction accounted for 11% of the PIM. This number is in line with a study on the prevalence of pharmacokinetic interactions in a psychiatric setting where 14.1% of such interactions were detected (46).

The “incorrect treatment duration” category concerned mostly benzodiazepines prescribed for more than 4 weeks. The long-term prescription of benzodiazepines, mainly for sleep problems and anxiety, is also common in the general population although it is associated with dependency and adverse events (47, 48).

We found an almost systematic long-term co-prescription of biperiden with antipsychotics. Most of the time, the indication for biperiden was not documented. Biperiden has not been proven to be effective for the indication of tardive dyskinesia, but might be prescribed to limit dystonia (49).

Adverse events were not included in the chosen definition of PIM. However, they were frequent in our study sample. In 75% of the hospitalization events, at least one AE was detected. This rate is a little lower than in another study on adults with ID in the Netherlands where 84% of patients had at least one AE (50).

Interestingly, we failed to correlate the number of PIM to age and ID severity. Other studies found that increased age and lower levels of ID were associated with higher risks of having PIM (6, 44). This might be due to the fact that we only had a small sample size and had lower age ranges than those found in other studies on PIM. No differences in prescription patterns were found between males and females. This is in line with what was found in the literature (44, 51, 52).

There was no significant difference between the mean number of drugs at entry and at discharge. However, the number of drugs evolved differently for each patient and adjustments in the prescriptions were made on an individual basis throughout hospitalization (e.g., drugs were deprescribed and replaced by others).

The improvement of the psychometric scale scores during the stay, despite poor prescription patterns, can probably be explained by specific behavioral approaches for patients with challenging behavior. This observation underscores the need for deprescribing. Deprescribing is the process of tapering, discontinuing, or withdrawing drugs in order to improve the patient's outcome or the cost-effectiveness of the treatment (53). Indeed, studies by de Kuijper et al. (54) showed that deprescribing of antipsychotics that were prescribed for challenging behavior in adults with ID led to an improvement in wellbeing and behavior (37).

### Limitations of the Study

Our study population is limited to patients hospitalized in a specialized mental health unit for ID, and even though different levels of ID were represented, the potential for generalization of our results is limited to this setting. Furthermore, some prescription practices, such as the lack of documentation or the frequent prescription of typical antipsychotics, seem to be local issues and cannot necessarily be generalized to other settings or other countries.

Another issue we encountered during the study was to obtain the consent of the patients' legal representatives. Indeed, among the screened 50 patients, only 13 legal representatives and 1 patient consented to participate, giving us a rejection rate of 72%. Among the main reasons was the difficulty to contact the legal representative rapidly and obtain a timely response, especially when those representatives were not the patient's parents. Too long delays led to patients being discharged before consent could be obtained.

Another limit of the study is the lack of documentation about indication or drug efficacy or adverse effects in the patient medical files in many cases. The patient files were then scrutinized for such information. When this information was definitely lacking, the prescription was considered as a PIM and classified as “lack of documentation.” Therefore, some diagnosis or good reasons for prescription may have been missed.

### Perspectives

Despite a growing literature challenging drug prescription and advocating for drug deprescription, namely, psychotropic drugs, in adults with ID, this study underscores that progress has to be made in clinical practice. The first step would be a systematic documentation of the treatment indication related to symptoms, as well as the intended treatment duration. Efficacy and adverse effects must also be routinely recorded and drug prescription questioned weekly. Safety and efficacy of prescription in patients with ID would be largely improved by the development of prescription guidelines or tools to guide physicians in the documentation, prescription, and deprescription process for patients with ID.

## Data Availability Statement

The raw data supporting the conclusions of this article will be made available by the authors, without undue reservation.

## Ethics Statement

The studies involving human participants were reviewed and approved by the Commission cantonale d'éthique de la recherche de Genève. The patients or their legal representatives provided their written informed consent to participate in this study.

## Author Contributions

SL, MK, MB, and FG formulated the research question and designed the study. SL wrote the protocol which was then revised by MK, MB, and FG. SL and FG collected and analyzed the data. SL wrote the paper. MK, MB, FG, JD, and J-MA reviewed the draft and contributed to the final version of the paper. All authors contributed to the article and approved the submitted version.

## Conflict of Interest

The authors declare that the research was conducted in the absence of any commercial or financial relationships that could be construed as a potential conflict of interest.
