# Supplementary material for: Prevalence of Polypharmacy and Inappropriate Medication in Adults With Intellectual Disabilities in a Hospital Setting in Switzerland
Source: Front Psychiatry. 2021 Jun 25;12:614825. doi: 10.3389/fpsyt.2021.614825 (PMC8267250; doi:10.3389/fpsyt.2021.614825)
Supplement: Supplementary file 1 [file Table_1.DOCX]

Appendix: List of all PIM recorded and number of corresponding occurrences

| **Lack of indication according to clinical context** | **35** | **Pharmacokinetic interaction** | **13** | **Pharmacodynamic interaction** | **12** |
| --- | --- | --- | --- | --- | --- |
| biperiden | 6 | CYP2D6 : metoprolol, quetiapine and risperidone | 1 | 3 anticholinergic drugs : hydroxyzine, clozapine, biperiden | 1 |
| esomeprazole | 7 | CYP2D6 : risperidone and aripiprazole | 1 | 3 anticholinergic drugs : levomepromazine, biperiden and butylscopolamine | 1 |
| lamotrigine | 3 | reduced glucuronidation by valproate : decreases lamotrigine clearance | 1 | 2 anticholinergic drugs : hydroxyzine and clozapine | 1 |
| venlafaxine | 1 | CYP2D6 : levomepromazine, venlafaxine and olanzapine | 1 | 2 anticholinergic drugs: biperiden and distigmine | 1 |
| levomepromazine | 5 | CYP2D6 : levomepromazine and aripiprazole | 1 | 2 anticholinergic drugs: atropine and biperiden | 1 |
| sertraline | 1 | CYP2B6, 2C9, 2C19, 3A4 : phenobarbital and lamotrigine, risperidone, midazolam, clonazepam, diazepam | 1 | 2 anticholinergic drugs: butylscopolamine and biperiden | 1 |
| pregabalin | 1 | CYP2D6 : risperidone, levomepromazine and sertraline | 1 | 2 anticholinergics : hydroxyzine + biperiden | 1 |
| clotiapine | 5 | CYP2D6 : venlafaxine and clozapine | 1 | Risk of QT prolongation : haloperidol and mirtazapine | 1 |
| trazodone | 1 | CYP2D6 : aripiprazole and duloxetine | 1 | Risk of QT prolongation in a patient with QT prolongation history : venlafaxine and clozapine | 1 |
| clomethiazole | 1 | CYP2D6 : metoclopramide, haloperidol, quetiapine, diphenydramine and mirtazapine | 1 | 3 anticholinergic drugs: diphenydramine, quetiapine and biperiden | 1 |
| paliperidone | 1 | CYP2D6: haloperidol, olanzapine and tramadol | 1 | Antagonism of metoclopramide (gastrokinetic) by anticholinergic drugs (biperiden and diphenhydramine) | 1 |
| atropine | 1 | CYP2C9: valproate and ibuprofene | 1 | Risk of EPS : metoclopramide, quetiapine and haloperidol | 1 |
| escitalopram | 1 | lithium and ibuprofen (increased serum lithium levels) | 1 | **Triplicate or more prescribing** | **5** |
| paroxetine | 1 | **Duplicate prescribing** | **23** | 3 antipsychotics: risperidone, quetiapine and clozapine | 1 |
| **Drug-disease interaction** | **2** | 2 benzodiazepines: flurazepam and clorazepate | 2 | 3 antipsychotics: aripiprazole, quetiapine and levomepromazine | 1 |
| Hallucinations and anticholinergic drugs | 1 | 2 benzodiazepines: lorazepam and flurazepam | 2 | 4 antiepileptics: lamotrigine, brivaracetam, clonazepam, phenobarbital | 1 |
| Metabolic syndrome and olanzapine | 1 | 2 benzodiazepines: midazolam and lorazepam | 1 | 3 antiepileptics : valproate, lamotrigine, rufinamide | 1 |
| **Lack of documentation** | **10** | 2 antipsychotics: aripiprazole and risperidone | 1 | 3 benzodiazepines : midazolam, diazepam, lorazepam | 1 |
| Increase of lamotrigine dosage without explanation | 1 | 2 antipsychotics: levomepromazine and olanzapine | 2 | **Incorrect use** | **4** |
| Elevated TSH for 2 months without any notes | 1 | 2 antipsychotics: levomepromazine and risperidone | 1 | clomethiazole 5x/day | 1 |
| Subtherapeutic concentration of paliperidone without any notes | 1 | 2 antipsychotics: clotiapine and quetiapine | 1 | Paliperidone abruptly withdrawn and resumed 2 days later because of withdrawal symptoms | 1 |
| Subtherapeutic concentration of haloperidol without any notes | 1 | 2 antipsychotics: paliperidone and haloperidol | 1 | Duloxetine abruptly withdrawn and resumed 5 days later because of withdrawal symptoms | 1 |
| Increase of aripiprazole dosage without any explanation | 1 | 2 antipsychotics: clotiapine and zuclopenthixol | 2 | Clotiapine abruptly withdrawn and resumed 2 weeks later | 1 |
| Subtherapeutic concentration of duloxetine without any notes | 1 | 2 antipsychotics: clotiapine and olanzapine | 1 | **Incorrect duration** | **10** |
| Enalapril | 1 | 2 antipsychotics: clotiapine and quetiapine | 1 | Benzodiazepines > 4 weeks : flurazepam | 2 |
| Simvastatine | 1 | 2 antipsychotics: levomepromazine and aripiprazole | 1 | Benzodiazepines > 4 weeks : clorazepate | 3 |
| Lamotrigine | 1 | 2 antipsychotics: haloperidol and quetiapine | 1 | Benzodiazepines > 4 weeks : lorazepam | 4 |
| Lithium | 1 | 2 antipsychotics: olanzapine and haloperidol | 1 | Benzodiazepines > 4 weeks : diazepam | 1 |
|  |  | 2 osmotic laxatives: macrogol and sorbitol | 2 |  |  |
|  |  | 2 osmotic laxatives: macrogol and lactitol | 2 |  |  |
|  |  | 2 stimulant laxatives: picosulfate and lubiprostone | 1 |  |  |
